# Supplementary figures and images for: Evolution of morphological and climatic adaptations in Veronica L. (Plantaginaceae)
Source: PeerJ. 2016 Aug 16;4:e2333. doi: 10.7717/peerj.2333 (PMC4991887; doi:10.7717/peerj.2333)

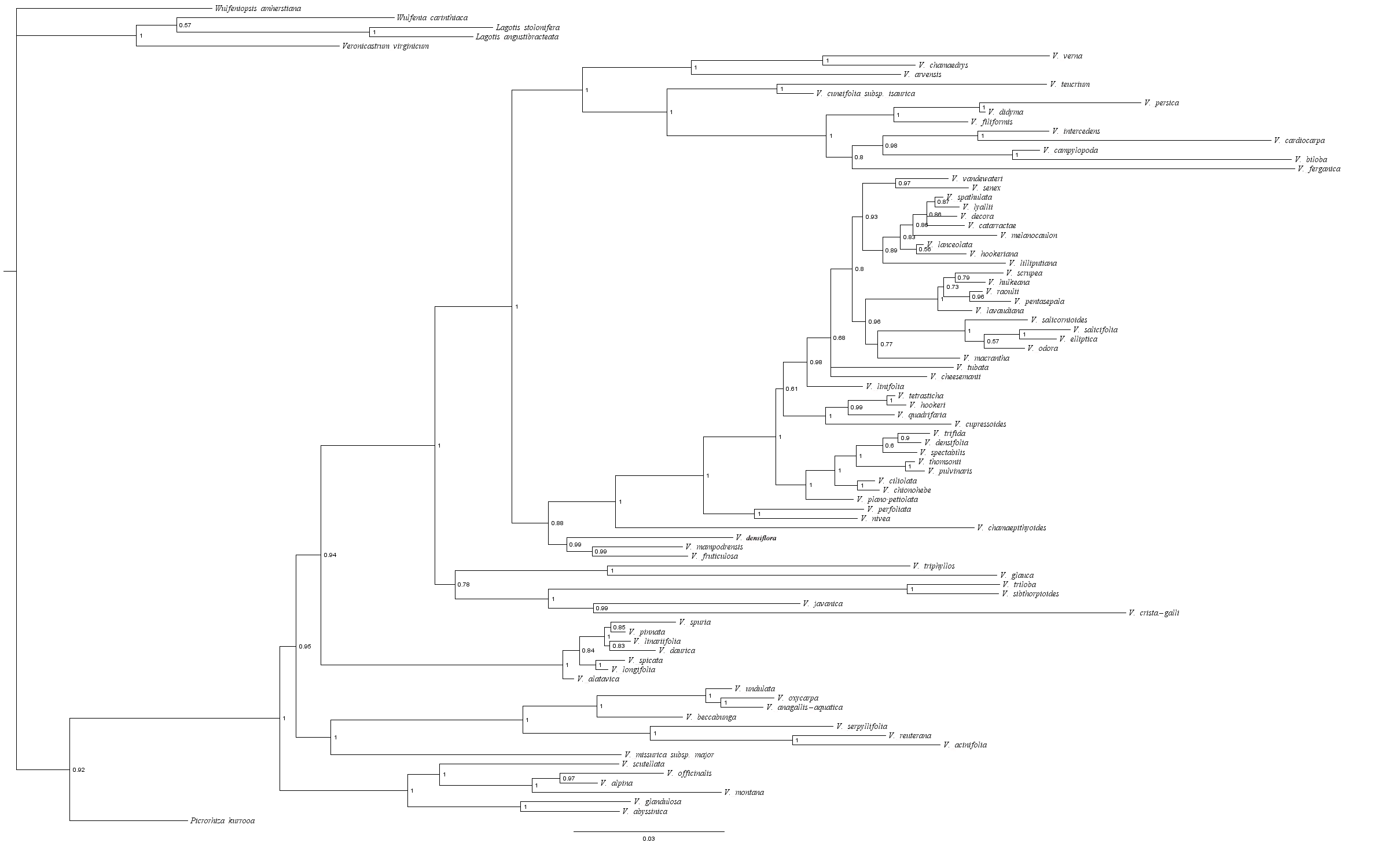

Supplement: Figrue S1 [file peerj-04-2333-s001.png]
