# Supplementary material for: Evolution of morphological and climatic adaptations in Veronica L. (Plantaginaceae)
Source: PeerJ. 2016 Aug 16;4:e2333. doi: 10.7717/peerj.2333 (PMC4991887; doi:10.7717/peerj.2333)
Supplement: Table S1 [file peerj-04-2333-s002.docx]

Supplementary Table 1.

GenBank Accession No. for specimens used in this study. Sequences newly sequenced for this study begin with KU and are in bold.

| Taxon name | ITS | *trnL-trnL-trnF* | *rps16* | *psbA-trnH* | Life history | Voucher |
| --- | --- | --- | --- | --- | --- | --- |
| *Lagotis angustibracteata* | AF313028 | AF486416 | AY218805 | FJ848085 | - | Miehe et al., 98-31710 GOET |
| *Lagotis stolonifera* | AF509814 | AF486415 | FJ848212 | FJ848086 | - | BG BONN 10240-30, BONN |
| *Picrorhiza kurrooa* | AF509813 | AF486414 | AY218806 | FJ848090 | - | R. McBeath 2214, K |
| *Veronicastrum virginicum* | AF313030 | AF486412 | AY218802 | FJ848087 | - | Chase s.n., K |
| *Wulfenia carinthiaca* | AF313025 | AF486409 | AY218804 | FJ848088 | - | Albach 74, WU |
| *Wulfeniopsis amherstiana* | FJ848064 | AF486411 | AY218808 | FJ848089 | - | Dickoree 13014, GOET |
| *V. abyssinica* | AF313009 | AF513350 | FJ848213 | FJ848092 | perennial | Fischer 728/98, BONN (ITS), E.Fischer 8060, BONN (others) |
| *V. acinifolia* | AF509798 | AF486399 | FJ848215 | FJ848098 | annual | M. Fischer s.n. (Lefkas), WU |
| *V. alatavica* | **KU047989** | **KU048030** | **KU048016** | **KU048003** | perennial | Wen-Jun Li, xy015-2 XJBI |
| *V. alpina* | AF313013 | AF486387 | FJ848214 | FJ848093 | perennial | Albach 184, WU |
| *V. anagallis-aquatica* | **KU047995** | **KU048036** | **KU048022** | **KU048009** | perennial | Jian-Cheng Wang, W20-3 XJBI |
| *V. arvensis* | AF313002 | AF486380 | FJ848224 | FJ848115 | annual | Albach 147, WU |
| *V. beccabunga* | AF313015 | AF486403 | AY218820 | FJ848099 | perennial | Albach 122, K |
| *V. biloba* | **KU047992** | **KU048033** | **KU048019** | **KU048006** | annual | Jian-Cheng Wang, W25-1 XJBI |
| *V. campylopoda* | AF486364 | AY673624 | AY218811 | FJ848117 | annual | Schönswetter and Tribsch 4152, WU (ITS, *rps*16,*CYC*2); Albach 656, WU (*trn*L-*trn*L-*trn*F, *psb*A-*trn*H, *rpo*B-*trn*C) |
| *V. cardiocarpa* | **KU047990** | **KU048031** | **KU048017** | **KU048004** | annual | Jian-Cheng Wang, W26-3 XJBI |
| *V. catarractae* | AY034859 | AY540887,AY540901 | FJ848253 | FJ848148 | perennial | Garnock-Jones 2403, CHR (ITS,*trn*L-*trn*L-*trn*F); Maichs.n., WELT SP086368 (*rps*16, *psb*A-*trn*H, *rpo*B-*trn*C, *CYC*2) |
| *V. chamaedrys* | AF313003 | AF486377 | AY218814 | FJ848116 | perennial | Albach 121, K |
| *V. chamaepithyoides* | AF509796 | AF511477/AF511478 | FJ848222 | FJ848112 | annual | UA 174, SALA |
| *V. cheesemanii* | FJ848078 | FJ848053 | FJ848254 | FJ848149 | perennial | Garnock-Jones 2587, WELTU |
| *V. chionohebe* | FJ848070 | FJ848044 | FJ848233 | FJ848127 | perennial | Bayly 1843, WELT SP084042 |
| *V. ciliolata* | FJ848067 | FJ848041 | FJ848230 | FJ848124 | perennial | Bayly 1812, WELT SP084019 |
| *V. crista-galli* | AF509799 | AF486367 | AY218816 | FJ848109 | annual | Dolmkanov 17.4.1983, TBS |
| *V. cuneifolia subsp. isaurica* | AF486354 | AF486372 | AY218804 | FJ848121 | perennial | L. Struwe 1409, WU |
| *V. cupressoides* | AF037378 | AY540880,AY540894 | FJ848251 | FJ848145 | perennial | Buxton 15.12.1989, CHR 512449 |
| *V. daurica* | AF313023 | AF511479/AF511480 | FJ848217 | FJ848102 | perennial | Albach s.n, WU |
| *V. decora* | AF229047 | AY540877 | FJ848255 | FJ848150 | perennial | Glenny and Wagstaff 95.08, CHR 512467 |
| *V. densiflora* | **KU047985** | **KU048026** | **KU048012** | **KU047999** | perennial | Jian-Cheng Wang, W43-3 XJBI |
| *V. densifolia* | FJ848068 | FJ848042 | FJ848231 | FJ848125 | perennial | Bayly 1779, WELT SP083990 (Australia) |
| *V. didyma* | AF509818 | AF486369 | FJ848227 | FJ848120 | annual | Albach 146, WU |
| *V. elliptica* | AF037393 | AY540883,AY540897 | FJ848242 | FJ848138 | perennial | Heenan 12.9.89, CHR 512484 |
| *V. ferganica* | **KU047984** | **KU048025** | **KU242588** | **KU047998** | annual | Jian-Cheng Wang, W14-3-2 XJBI |
| *V. filiformis* | AF486363 | AF486368 | FJ848225 | FJ848118 | perennial | Albach 298, WU (ITS, *trn*L-*trn*L-*trn*F); Albach 858, WU (*rps*16,*psb*A-*trn*H,*rpo*B-*trn*C) |
| *V. fruticulosa* | AF313004 | AF486383 | AY218812 | FJ848113 | perennial | Albach 71, BONN |
| *V. glandulosa* | AF313008 | AF486394 | AY218822 | FJ848094 | perennial | Fischer 713/98, WU |
| *V. glauca* | AF313006 | AF486395 | FJ848220 | FJ848108 | annual | M. Fischer 9, 7.4.1999, WU |
| *V. hookeri* | FJ848077 | FJ848050 | FJ848250 | FJ848144 | perennial | Bayly 1733, WELT SP083954 |
| *V. hookeriana* | FJ848079 | FJ848054 | FJ848256 | FJ848151 | perennial | Thorsen s.n., WELT SP086389 |
| *V. hulkeana* | FJ848075 | n.a. | FJ848238 | FJ848132 | perennial | Garnock-Jones 2593, WELTU 20205 |
| *V. intercedens* | AY673609 | AY673628 | FJ848226 | FJ848119 | annual | Albach 666, WU |
| *V. javanica* | AY540867 | AY540872 | FJ848219 | FJ848106 | annual | Fries et al. 2016, BR (ITS, *trn*L-*trn*L-*trn*F); Garnock-Jones s.n., cult from seeds of PGJ 2621, WELT SP086369 (*rps*16, *psb*A-*trn*H, *rpo*B-*trn*C) |
| *V. lanceolata* | n.a. | FJ848055 | FJ848257 | FJ848152 | perennial | Garnock-Jones 2632, WELT SP086371 |
| *V. lavaudiana* | AF229043 | n.a. | FJ848239 | FJ848133 | perennial | Molloy 8.9.82, CHR 512487 |
| *V. lilliputiana* | AF037394 | FJ848052 | AY21813 | FJ848147 | perennial | Garnock-Jones Aug 1990, CHR 512446 |
| *V. linariifolia* | **KU047993** | **KU048034** | **KU048020** | **KU048007** | perennial | Jian-Cheng Wang, W28-3 XJBI |
| *V. linifolia* | FJ848080 | FJ848056 | FJ848258 | FJ848153 | perennial | Molloy and Druce, 22.02.1991, CHR 470347 |
| *V. longifolia* | AF313021 | AF486407 | AY218818 | FJ848103 | perennial | Albach 66, BONN |
| *V. lyallii* | FJ848081 | FJ848057 | FJ848259 | FJ848154 | perennial | Bayly 585, WELT SP080859/A |
| *V. macrantha* | AY 034853 | FJ848048 | FJ848244 | FJ848137 | perennial | Glenny and Wagstaff 95.09, CHR 512468 (*V*. *macrantha* 2) |
| *V. mampodrensis* | DQ227331 | DQ227337 | FJ848223 | FJ848114 | perennial | Martínez-Ortega 713, SALA |
| *V. melanocaulon* | AF037396 | FJ848058 | FJ848260 | FJ848155 | perennial | Williams 15.04.1976, CHR 512456 |
| *V. missurica subsp. major* | AF313019 | AF486397 | AY218819 | FJ848105 | perennial | Albach 124, K |
| *V. montana* | AF313014 | AF486388 | AY218824 | FJ848095 | perennial | Albach 151, WU |
| *V. nivea* | AF037382 | AY540890,AY540903 | FJ848229 | FJ848123 | perennial | Wagstaff 94.105, CHR 512486 |
| *V. odora* | AF037388 | AY540882,AY540896 | FJ848245 | FJ848139 | perennial | Garnock-Jones 4.12.90, CHR 512441 |
| *V. officinalis* | AF313012 | AF486391 | n.a. | FJ848096 | perennial | Albach and Chase 114, K |
| *V. oxycarpa* | **KU047987** | **KU048028** | **KU048014** | **KU048001** | perennial | Lei Wang, L4-1 XJBI |
| *V. pentasepala* | FJ848076 | n.a. | FJ848240 | FJ848134 | perennial | Garnock-Jones 2589, WELTU |
| *V. perfoliata* | FJ848066 | FJ848040 | FJ848228 | FJ848122 | perennial | NSW 502261 |
| *V. persica* | **KU047996** | **KU048037** | **KU048023** | **KU048010** | annual | Jian-Cheng Wang, W14 XJBI |
| *V. pinnata* | **KU047997** | **KU048038** | **KU048024** | **KU048011** | perennial | Yan Li, LY14-1(1) XJBI |
| *V. plano-petiolata* | AF229050 | FJ848059 | FJ848261 | FJ848156 | perennial | Simpson s.n., CHR 512626 (ITS); Garnock-Jones 2606a, WELTU 20207 (*trn*L-*trn*L-*trn*F,*rps*16, *psb*A-*trn*H, *rpo*B-*trn*C, *CYC*2) |
| *V. pulvinaris* | FJ848072 | FJ848046 | FJ848235 | FJ848129 | perennial | Glenny 9093, WELT SP084074 |
| *V. quadrifaria* | AF037377 | AY540886,AY540900 | FJ848249 | FJ848143 | perennial | Heenan and Garnock-Jones 1999, CHR 512472 |
| *V. raoulii* | AF037380 | AY540885,AY540899 | FJ848241 | FJ848135 | perennial | Decourtye 22.2.86, CHR 512459 |
| *V. reuterana* | AY540866 | AY486447 | FJ848216 | FJ848100 | annual | Albach 676, WU |
| *V. salicifolia* | AF037386 | FJ848049 | FJ848248 | FJ848142 | perennial | Buxton 1992, WELTU 16898 (South America) |
| *V. salicornioides* | AF069465 | AY540879,AY540893 | FJ848246 | FJ848140 | perennial | Hair 26.10.6, CHR 512475 |
| *V. scrupea* | FJ848074 | n.a. | FJ848237 | FJ848131 | perennial | Garnock-Jones 2592, WELTU 20206 |
| *V. scutellata* | AF509805 | AF486393 | AY218823 | FJ848097 | perennial | C. Dobes 7026, WU |
| *V. senex* | FJ848082 | FJ848060 | FJ848262 | FJ848157 | perennial | Garnock-Jones 2360, WELT SP086370 |
| *V. serpyllifolia* | AF313017 | AF486400 | AY218821 | FJ848101 | perennial | Albach 64, WU |
| *V. sibthorpioides* | AY850099 | AY540876 | FJ848221 | FJ848110 | annual | Martínez-Ortega 831, SALA |
| *V. spathulata* | AF229051 | FJ848061 | FJ848263 | FJ848158 | perennial | Garnock-Jones 2263, WELTU 16860 |
| *V. spectabilis* | AF229044 | FJ848062 | FJ848264 | FJ848159 | perennial | Garnock-Jones and Malcolm 2039, CHR 470104 |
| *V. spicata* | AF313022 | AF486405 | FJ848218 | FJ848104 | perennial | Albach 65, BONN (ITS,*trn*L-*trn*L-*trn*F); Andy Jones s.n. K 5.3 3 (*rps*16, *psb*A-*trn*H, *rpo*B-*trn*C) |
| *V. spuria* | **KU047988** | **KU048029** | **KU048015** | **KU048002** | perennial | Jian-Cheng Wang, W34-1 XJBI |
| *V. tetrasticha* | AY034866,AY034867 | FJ848051 | FJ848252 | FJ848146 | perennial | Druce 20.10,1989, CHR 512451 |
| *V. teucrium* | **KU047986** | **KU048027** | **KU048013** | **KU048000** | perennial | Jian-Cheng Wang, W37-1 XJBI |
| *V. thomsonii* | FJ848073 | FJ848047 | FJ848236 | FJ848130 | perennial | Bayly 1651, WELT SP083901 (*V*.*thomsonii* 1) |
| *V. trifida* | FJ848083 | FJ848063 | FJ848265 | FJ848160 | perennial | Bayly 1841, WELT SP084041 |
| *V. triloba* | AF509804 | AF513333 | AY218815 | FJ848111 | annual | Albach 242, WU |
| *V. triphyllos* | FJ848065 | FJ848039 | AY218817 | FJ848107 | annual | Albach 832, WU (ITS, *trn*L-*trn*L-*trn*F, *psb*A-*trn*H, *rpo*B-*trn*C), Albach 244, WU (*rps*16) |
| *V. tubata* | AY540870 | AY540874 | n.a. | n.a. | perennial | M.M.J. v. Balgooy 566, K |
| *V. undulata* | **KU047994** | **KU048035** | **KU048021** | **KU048008** | perennial | Jian-Cheng Wang, W20-6 XJBI |
| *V. vandewateri* | FJ848084 | AF486381 | AY21813 | FJ848161 | perennial | Barker 59, K |
| *V. verna* | **KU047991** | **KU048032** | **KU048018** | **KU048005** | annual | Jian-Cheng Wang, W25-4 XJBI |
