# Supplementary material for: Evolution of morphological and climatic adaptations in Veronica L. (Plantaginaceae)
Source: PeerJ. 2016 Aug 16;4:e2333. doi: 10.7717/peerj.2333 (PMC4991887; doi:10.7717/peerj.2333)
Supplement: Table S2 [file peerj-04-2333-s003.docx]

Supplementary Table 2.

Characters and character states used in morphometric analysis

| Character | Coding |
| --- | --- |
| Leaf length | >20 mm (0), ≤20 mm (1) |
| Leaf width | ≤30 mm (0), >30 mm (1) |
| Bract shape | oblong, linear to lanceolate (0), ovate-elliptic to lobate (1) |
| Bract length | >7 mm (0), ≤7 mm (1) |
| Corolla shape | tubular (0), rotate (1) |
| Corolla diameter | >6 mm (0), ≤6 mm (1) |
| Stamen length | >5 mm (0), ≤5 mm (1) |
| Capsule apex | non-emarginate or shallowly emarginate (0), deeply emarginate (1) |
| Style length | >8 mm (0), ≤8 mm (1) |
