# Supplementary material for: Evolution of morphological and climatic adaptations in Veronica L. (Plantaginaceae)
Source: PeerJ. 2016 Aug 16;4:e2333. doi: 10.7717/peerj.2333 (PMC4991887; doi:10.7717/peerj.2333)
Supplement: Table S3 [file peerj-04-2333-s006.docx]

Supplementary Table 3.

Data matrix used in morphometric analysis of the genus Veronica.

| Species | Leaf length | Leaf width | Bract shape | Bract length | Corolla shape | Corolla diameter | Stamen length | Style length |
| --- | --- | --- | --- | --- | --- | --- | --- | --- |
| *V. abyssinica* | 1 | 1 | 0 | 1 | 1 | 0 | 1 | 1 |
| *V. acinifolia* | 1 | 1 | 1 | 1 | 1 | 1 | 1 | 1 |
| *V. alatavica* | 0 | 1 | 0 | 1 | 0 | 1 | 0 | 1 |
| *V. alpina* | 0 | 1 | 0 | 0 | 0 | 0 | 1 | 1 |
| *V. anagallis. aquatica* | 0 | 1 | 0 | 1 | 1 | 1 | 1 | 1 |
| *V. arvensis* | 1 | 1 | 0 | 1 | 1 | 1 | 1 | 1 |
| *V. beccabunga* | 1 | 1 | 0 | 1 | 1 | 1 | 1 | 1 |
| *V. biloba* | 1 | 1 | 1 | 0 | 1 | 1 | 1 | 1 |
| *V. campylopoda* | 1 | 1 | 0 | 1 | 1 | 1 | 1 | 1 |
| *V. cardiocarpa* | 0 | 1 | 1 | 0 | 1 | 1 | 1 | 1 |
| *V. catarractae* | 0 | 1 | 0 | 1 | 1 | 0 | 0 | 1 |
| *V. chamaedrys* | 0 | 1 | 0 | 1 | 1 | 0 | 0 | 1 |
| *V. chamaepithyoides* | 1 | 1 | 1 | 0 | 1 | 1 | 1 | 1 |
| *V. cheesemanii* | 1 | 1 | 1 | 1 | 0 | 0 | 1 | 1 |
| *V. chionohebe* | 1 | 1 | 0 | 1 | 0 | 1 | 1 | 1 |
| *V. ciliolata* | 1 | 1 | 0 | 1 | 0 | 1 | 1 | 1 |
| *V. crista. galli* | 0 | 1 | 1 | 0 | 1 | 0 | 0 | 1 |
| *V. cuneifolia subsp. isaurica* | 1 | 1 | 0 | 1 | 1 | 0 | 1 | 1 |
| *V. cupressoides* | 1 | 1 | 0 | 1 | 0 | 1 | 1 | 1 |
| *V. daurica* | 0 | 1 | 0 | 1 | 0 | 0 | 0 | 0 |
| *V. decora* | 1 | 1 | 0 | 1 | 1 | 0 | 0 | 1 |
| *V. densiflora* | 1 | 1 | 1 | 0 | 1 | 1 | 0 | 1 |
| *V. densifolia* | 1 | 1 | 0 | 1 | 0 | 0 | 0 | 1 |
| *V. didyma* | 1 | 1 | 1 | 0 | 1 | 1 | 1 | 1 |
| *V. elliptica* | 0 | 1 | 0 | 1 | 0 | 0 | 0 | 1 |
| *V. ferganica* | 1 | 1 | 1 | 1 | 1 | 1 | 1 | 1 |
| *V. filiformis* | 1 | 1 | 0 | 1 | 1 | 0 | 0 | 1 |
| *V. fruticulosa* | 0 | 1 | 0 | 1 | 1 | 0 | 0 | 1 |
| *V. glandulosa* | 0 | 1 | 1 | 0 | 1 | 0 | 1 | 1 |
| *V. glauca* | 1 | 1 | 0 | 1 | 1 | 0 | 1 | 1 |
| *V. hookeri* | 1 | 1 | 0 | 1 | 0 | 1 | 1 | 1 |
| *V. hookeriana* | 1 | 1 | 0 | 1 | 1 | 0 | 0 | 1 |
| *V. hulkeana* | 0 | 1 | 0 | 1 | 1 | 0 | 1 | 1 |
| *V. intercedens* | 1 | 1 | 0 | 1 | 1 | 1 | 1 | 1 |
| *V. javanica* | 0 | 1 | 0 | 1 | 1 | 1 | 1 | 1 |
| *V. lanceolata* | 0 | 1 | 0 | 1 | 1 | 0 | 1 | 1 |
| *V. lavaudiana* | 0 | 1 | 0 | 1 | 0 | 0 | 1 | 1 |
| *V. lilliputiana* | 1 | 1 | 1 | 1 | 1 | 0 | 0 | 1 |
| *V. linariifolia* | 0 | 1 | 0 | 1 | 0 | 1 | 0 | 1 |
| *V. linifolia* | 0 | 1 | 0 | 0 | 1 | 0 | 0 | 0 |
| *V. longifolia* | 0 | 1 | 0 | 1 | 0 | 1 | 0 | 1 |
| *V. lyallii* | 1 | 1 | 0 | 1 | 1 | 0 | 1 | 1 |
| *V. macrantha* | 0 | 1 | 0 | 1 | 0 | 0 | 1 | 1 |
| *V. mampodrensis* | 1 | 1 | 0 | 0 | 1 | 0 | 1 | 1 |
| *V. melanocaulon* | 0 | 1 | 0 | 1 | 1 | 0 | 1 | 1 |
| *V. missurica subsp. major* | 0 | 0 | 0 | 1 | 0 | 1 | 0 | 1 |
| *V. montana* | 0 | 1 | 0 | 1 | 1 | 0 | 1 | 1 |
| *V. nivea* | 0 | 1 | 0 | 1 | 1 | 0 | 0 | 1 |
| *V. odora* | 0 | 1 | 0 | 1 | 0 | 0 | 0 | 0 |
| *V. officinalis* | 0 | 1 | 0 | 1 | 1 | 1 | 1 | 1 |
| *V. oxycarpa* | 0 | 1 | 0 | 1 | 1 | 1 | 1 | 1 |
| *V. pentasepala* | 0 | 1 | 0 | 1 | 0 | 0 | 1 | 1 |
| *V. perfoliata* | 0 | 0 | 0 | 1 | 1 | 0 | 0 | 1 |
| *V. persica* | 1 | 1 | 1 | 0 | 1 | 1 | 1 | 1 |
| *V. pinnata* | 0 | 1 | 0 | 1 | 0 | 1 | 1 | 1 |
| *V. plano-petiolata* | 1 | 1 | 0 | 0 | 0 | 0 | 1 | 1 |
| *V. pulvinaris* | 1 | 1 | 0 | 1 | 0 | 1 | 0 | 1 |
| *V. quadrifaria* | 1 | 1 | 1 | 1 | 0 | 1 | 1 | 1 |
| *V. raoulii* | 0 | 1 | 0 | 1 | 0 | 1 | 0 | 1 |
| *V. reuterana* | 1 | 1 | 0 | 1 | 1 | 1 | 1 | 1 |
| *V. salicifolia* | 1 | 1 | 0 | 1 | 0 | 0 | 0 | 1 |
| *V. salicornioides* | 1 | 1 | 1 | 1 | 0 | 0 | 0 | 1 |
| *V. scrupea* | 1 | 1 | 0 | 1 | 0 | 0 | 1 | 1 |
| *V. scutellata* | 0 | 1 | 0 | 1 | 1 | 1 | 1 | 1 |
| *V. senex* | 0 | 1 | 0 | 0 | 1 | 0 | 1 | 1 |
| *V. serpyllifolia* | 1 | 1 | 1 | 0 | 1 | 1 | 1 | 1 |
| *V. sibthorpioides* | 1 | 1 | 1 | 0 | 1 | 1 | 1 | 1 |
| *V. spathulata* | 1 | 1 | 1 | 1 | 0 | 0 | 1 | 1 |
| *V. spectabilis* | 1 | 1 | 1 | 0 | 0 | 0 | 1 | 1 |
| *V. spicata* | 0 | 1 | 0 | 1 | 0 | 1 | 0 | 1 |
| *V. spuria* | 0 | 1 | 0 | 1 | 0 | 1 | 1 | 1 |
| *V. tetrasticha* | 1 | 1 | 1 | 1 | 0 | 0 | 1 | 1 |
| *V. teucrium* | 0 | 1 | 0 | 1 | 1 | 0 | 1 | 1 |
| *V. thomsonii* | 1 | 1 | 1 | 1 | 0 | 1 | 1 | 1 |
| *V. trifida* | 1 | 1 | 1 | 1 | 0 | 0 | 0 | 1 |
| *V. triloba* | 1 | 1 | 1 | 0 | 1 | 1 | 1 | 1 |
| *V. triphyllos* | 1 | 1 | 1 | 1 | 1 | 1 | 1 | 1 |
| *V. tubata* | 1 | 1 | 1 | 1 | 0 | 0 | 1 | 1 |
| *V. undulata* | 0 | 1 | 0 | 1 | 1 | 1 | 1 | 1 |
| *V. vandewateri* | 1 | 1 | 1 | 1 | 0 | 0 | 1 | 1 |
| *V. verna* | 1 | 1 | 0 | 1 | 1 | 1 | 1 | 1 |
